# Dendritic cell‐targeted delivery of antigens using extracellular vesicles for anti‐cancer immunotherapy

**DOI:** 10.1111/cpr.13622

**Published:** 2024-03-20

**Authors:** Xuan T. T. Dang, Cao Dai Phung, Claudine Ming Hui Lim, Migara Kavishka Jayasinghe, Jorgen Ang, Thai Tran, Herbert Schwarz, Minh T. N. Le

**Affiliations:** ^1^ Department of Pharmacology, Yong Loo Lin School of Medicine National University of Singapore Singapore Singapore; ^2^ Institute for Digital Medicine, Yong Loo Lin School of Medicine National University of Singapore Singapore Singapore; ^3^ School of Applied Science Republic Polytechnic Woodlands Singapore; ^4^ Department of Physiology, Yong Loo Lin School of Medicine National University of Singapore Singapore Singapore; ^5^ Infectious Disease Translational Research Program National University of Singapore Singapore Singapore; ^6^ Immunology Programme National University of Singapore Singapore Singapore; ^7^ Department of Surgery, Yong Loo Lin School of Medicine National University of Singapore Singapore Singapore; ^8^ Institute of Molecular and Cell Biology Agency for Science, Technology, Technology and Research Singapore Singapore

## Abstract

Neoantigen delivery using extracellular vesicles (EVs) has gained extensive interest in recent years. EVs derived from tumour cells or immune cells have been used to deliver tumour antigens or antitumor stimulation signals. However, potential DNA contamination from the host cell and the cost of large‐scale EV production hinder their therapeutic applications in clinical settings. Here, we develop an antigen delivery platform for cancer vaccines from red blood cell‐derived EVs (RBCEVs) targeting splenic DEC‐205^+^ dendritic cells (DCs) to boost the antitumor effect. By loading ovalbumin (OVA) protein onto RBCEVs and delivering the protein to DCs, we were able to stimulate and present antigenic OVA peptide onto major histocompatibility complex (MHC) class I, subsequently priming activated antigen‐reactive T cells. Importantly, targeted delivery of OVA using RBCEVs engineered with anti‐DEC‐205 antibody robustly enhanced antigen presentation of DCs and T cell activation. This platform is potentially useful for producing personalised cancer vaccines in clinical settings.

## Graphical abstract

Red blood cell‐derived extracellular vesicles (RBCEVs) are loaded with protein antigen and are surface‐modified with DEC205‐targeting antibodies. The antigen‐loaded, DEC205‐targeting RBCEVs are taken up by dendritic cells and antigens are processed. Via DEC205‐mediated endocytosis, the antigens are digested and presented on MHC class I, which, in turn, activate CD8^+^ T cells. This mode of action improves T cell proliferation and activation while providing targeting effect towards secondary lymphoid organs in mice.

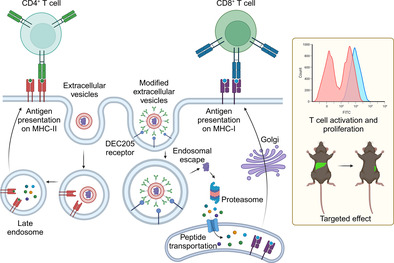

## 1 BACKGROUND

By utilising the host's immune system to target and attack tumour cells, cancer immunotherapy exhibits high specificity and low toxicity to eliminate cancer cells and prevent their relapse.^1^ The current cancer immunotherapeutic approaches, such as cancer vaccines, immune checkpoint inhibitors and adoptive cell therapy have shown positive clinical outcomes in treating various types of cancers.^2^ Among these, cancer vaccines demonstrate high specificity to fight against tumour cells with low side effects.^3^ An effective cancer vaccine often comprises tumour antigens, adjuvants and possibly the delivery carrier.^4^

To date, all of the existing cancer vaccines aim to trigger a specific antitumor immune response through dendritic cell (DC) activity.^5^ Vaccination approaches using ex vivo antigen‐loaded and matured monocyte‐derived DCs have demonstrated to augment strong and durable cytotoxic T lymphocyte (CTL) responses against the tumours.^6^ However, despite considerable interest and ample research, this approach remains time‐consuming and costly. Alternatively, antigen‐loaded particle‐based delivery systems, such as polymeric nanoparticles, liposomes, virosomes, extracellular vesicles (EVs) and bioconjugates are yielding promising results in the field of vaccine development. This is probably because they are generally easily recognised and ingested by DCs, and because they can be engineered to carry a wide range of therapeutic agents and targeting moieties to improve DC targeting.^7^

EVs are naturally released, cell‐derived phospholipid bilayer‐enclosed nanoparticles. The source of the EVs can affect the EV's characteristics, biodistribution and functions.^8^, ^9^ EVs derived from tumour cells or immune cells have been employed as cancer vaccine candidates in cancer immunotherapy, as they carry neoantigens for DCs stimulation, or they can activate T cell immune response on their own as cell‐free antitumor vesicles.^10^, ^11^ EVs can also be engineered with enhanced targeting ability to deliver antigens to DCs specifically.^12^ However, EVs derived from nucleated cells possess the risk of horizontal DNA transfer.^13^ Alternatively, the use of red blood cells (RBCs)‐derived EVs (RBCEVs) may reduce the risk of genomic DNA transfer as mature RBCs are enucleated. RBCEVs are also resistant to damage even after multiple freeze–thaw cycles and can be stored at a low temperature for a long time.^14^ Our research group has established a method of large‐scale production and purification of RBCEVs with a yield of 10^13^–10^14^ EVs per blood unit.^14^ We have successfully developed RBCEVs as a delivery system for multiple therapeutic agents, including RNA, DNA and chemotherapy.^14^ Furthermore, we reported a simple and efficient method to conjugate RBCEVs with targeting peptides, antibodies and nanobodies.^15^, ^16^, ^17^, ^18^ Thus, RBCEVs might be a suitable platform for nanovaccine development owing to their safety and non‐immunogenicity. Their production is scalable and economical as the EVs can be obtained directly from the blood, which is readily available.

C‐type lectin receptors (CLRs) are a group of proteins that recognise specific carbohydrate structures on pathogens and mediate immune responses. DCs express various CLRs that can be exploited for targeted delivery of antigens or adjuvants, such as mannose receptor, DEC‐205, DC‐SIGN, Langerin, DCIR2 and Clec9A.^19^, ^20^ These CLRs differ in their ligand specificity, expression pattern and signalling pathways. By modulating the CLR‐mediated signalling in DCs, different types of T cell responses can be induced or modulated. DEC‐205 is expressed by CD8α^+^ DCs, which are specialised in cross‐presentation and initiation of cellular immune responses.^21^ Targeting vaccine antigens to the DEC‐205^+^ DCs has proved an effective way to induce CTLs and antibody responses.^22^, ^23^

In this paper, we describe a method for antigen delivery using RBCEVs. We demonstrate that RBCEVs can be loaded with either mRNAs, long peptides or proteins and delivered as bioactive cargoes to DCs. RBCEVs can also be modified on the surface with Fc‐binding peptide (FcBP) and anti‐DEC‐205 antibody (αDECab) to improve targeting ability and enhance antigen presentation of DEC‐205^+^ DCs. We showed that RBCEVs‐mediated antigen delivery to DCs via DEC‐205 receptor could express higher antigen onto MHC class I (MHC‐I) molecules, subsequently stimulating cytotoxic CD8^+^ T cell proliferation and activation. Moreover, this platform could be easily applicable to target other molecules by simply varying the antibody of interest.

## 2 RESULTS

### 2.1 RBCEVs deliver loaded mRNA, peptide and protein cargo to DCs

**FIGURE 1:**
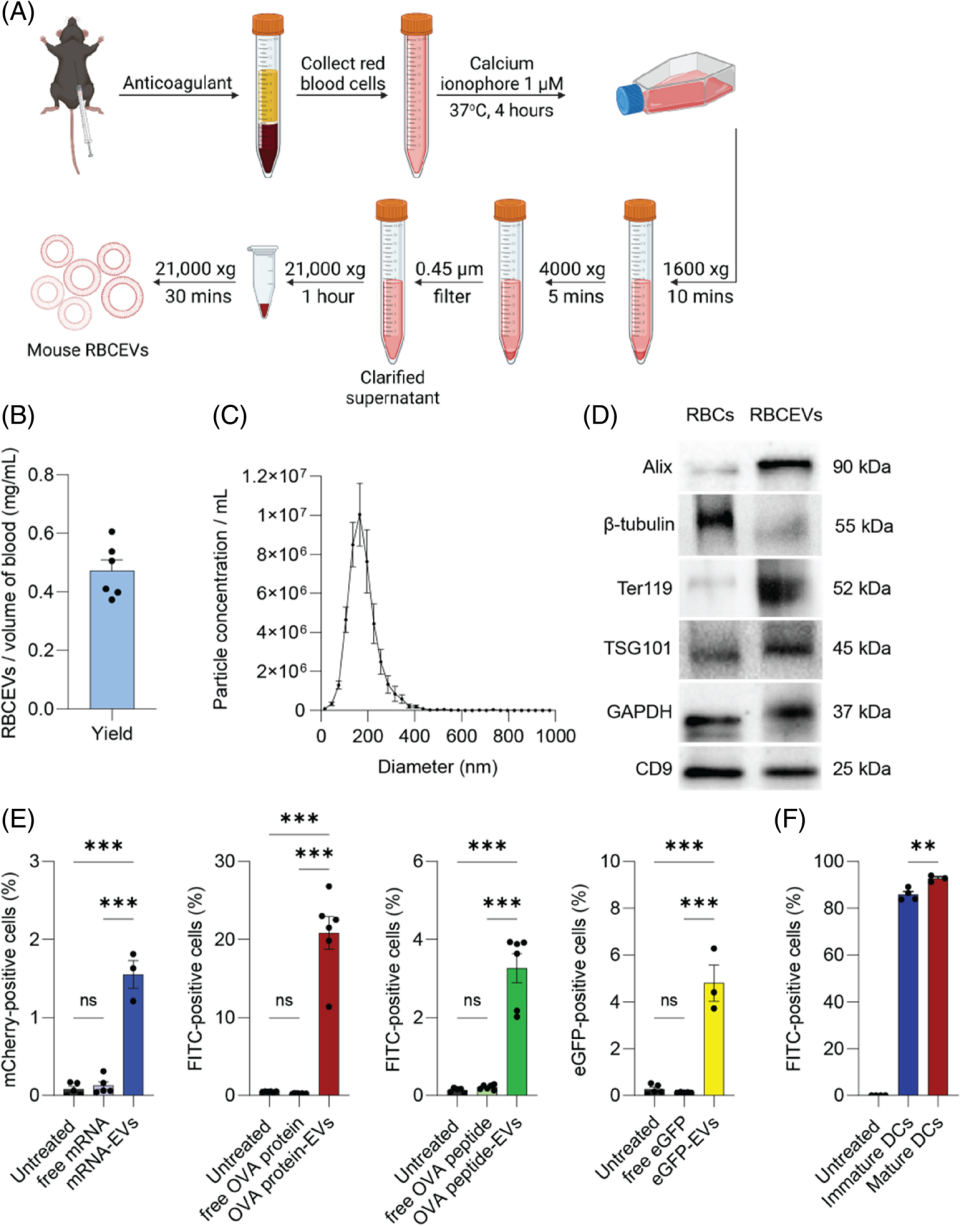
Red blood cell‐derived extracellular vesicles (RBCEVs) deliver protein, peptide and mRNA to dendritic cells at different efficiency. (A) Generation of mouse RBCEVs from mouse blood, where RBCs were treated with calcium ionophore to increase extracellular vesicle release. (B) Average yield of RBCEVs per mL of collected mouse blood (*n* = 6 independent batches). (C) Average size distribution of mouse RBCEVs determined by nanoparticle tracking analysis (*n* = 3). (D) Western blot analysis of proteins in mouse RBCs and purified mouse RBCEVs. (E) Flow cytometric analysis of DC2.4 cells treated with mouse RBCEVs loaded with mCherry mRNA, FITC‐labelled ovalbumin (OVA) protein, FITC‐labelled OVA long peptide (20 amino acids) or eGFP protein (*n* = 3–6 biological repeats). (F) Flow cytometric analysis of human monocyte‐derived conventional dendritic cells (mo‐cDCs) treated with human RBCEVs loaded with FITC‐labelled OVA protein. All graphs present mean ± SEM.**p < 0.01; ***p < 0.001, determined by one‐way ANOVA with Tukey's post‐hoc test.

We first induced RBCEV production from mouse RBCs collected from C57BL/6 mice and purified the released EVs using sequential centrifugation and filtration to remove cell debris and protein contamination (Figure 1A). On average, we could purify 0.465 mg of mouse RBCEVs per mL of collected mouse blood (Figure 1B). Purified mouse RBCEVs were heterogeneous, with an average size of 160 nm (Figure 1C). Purified RBCEVs were enriched in EV markers such as CD9, TSG101 and Alix compared with mouse RBCs (Figure 1D). Ter119, a marker for mouse RBCs, were enriched in RBCEVs, while β‐tubulin, a marker for cytoskeleton, was more abundant in RBCs (Figure 1D). This observation underscores that, at the same level of protein input, RBCEVs contain more membrane and less cytoskeleton than RBCs.

In this study, we selected OVA as a model antigen. We examined the ability of RBCEVs to deliver different types of payloads including mRNA, protein, or long peptide, to a murine DC cell line (DC2.4 cells). The antigens were loaded onto EVs using REG‐1 as previously described.^18^ Flow cytometry analysis showed that mouse RBCEVs could deliver mRNA, OVA protein, or long synthetic OVA peptide into targeted cells at a much higher efficiency compared to free antigens, with transfection efficiencies of 1.6%, 20.8% and 2.7%, respectively (Figure 1E). As the transfection efficiency of OVA protein was the highest among tested antigens, we continued to use OVA protein in the subsequent experiments. We also performed the same experiments with human‐derived RBCEVs and found that human RBCEVs can deliver FITC‐OVA protein into human immature and mature DCs at a transfection efficiency of 90%–93% (Figure 1F), indicating the translational potential of this platform for clinical applications.

### 2.2 Functionalisation of RBCEVs with anti‐DEC‐205 antibody improved cellular uptake of RBCEVs and increased protein delivery into DC2.4 cells

**FIGURE 2:**
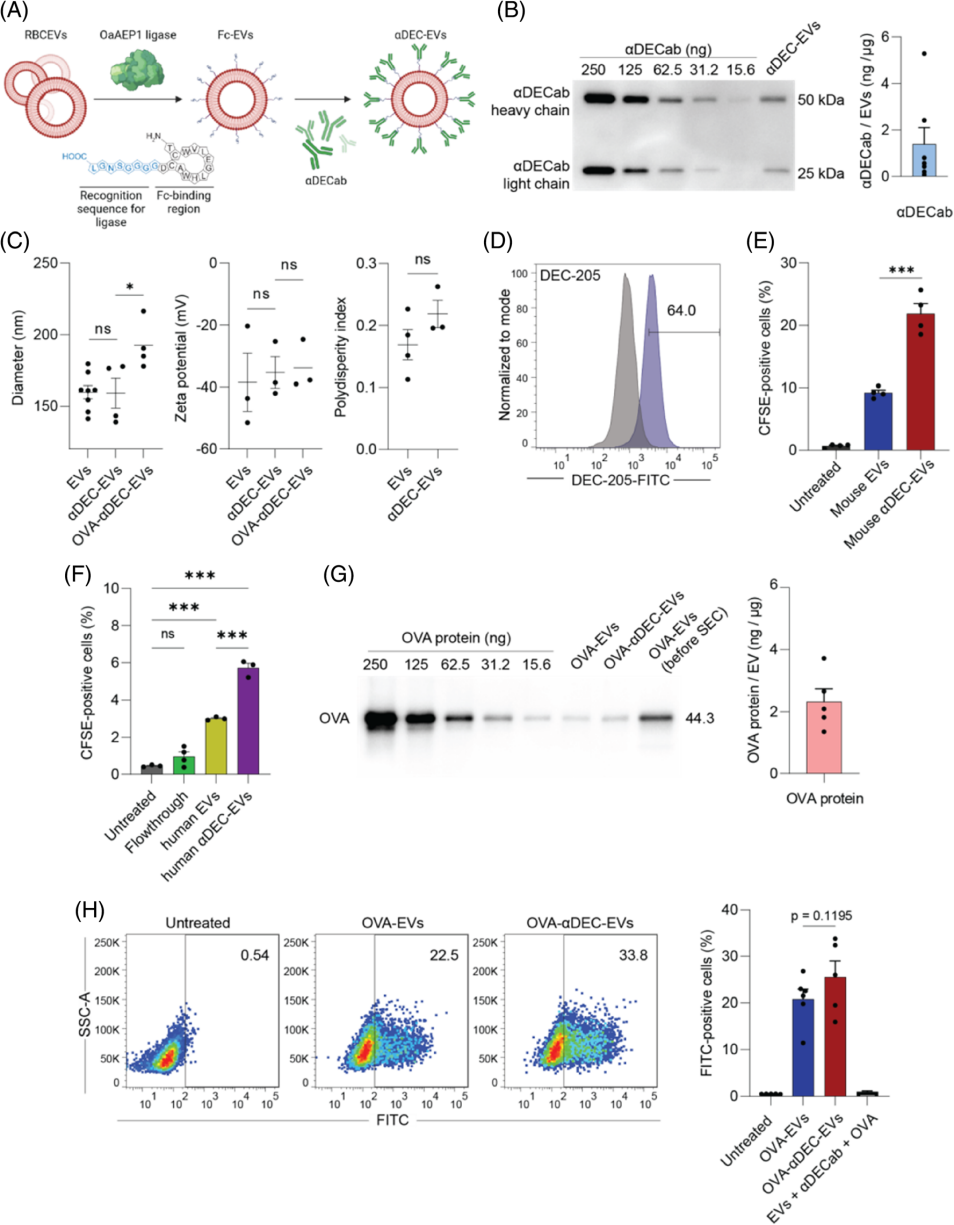
Modifying mouse red blood cell‐derived extracellular vesicles (RBCEVs) with αDECab improved cellular uptake of EVs in DC2.4 cells. (A) Schematic illustration of the ligation‐conjugation method for surface modification of RBCEVs. OaAEP1 ligase mediates a covalent ligation between NGL at the C‐terminal of the FcBP with the N‐terminal of EV proteins. (B) Western blot analysis and quantification of αDECab conjugation onto RBCEVs (DEC‐targeting EVs, αDEC‐EVs) showing bands corresponding to both heavy chain (50 kDa) and light chain (25 kDa). (C) Average diameter (*n* = 4–6), zeta potential (*n* = 3), and polydispersity index of mouse RBCEVs and their modified and loaded derivatives, measured by NTA and dynamic light scattering method, respectively. (D) Flow cytometric analysis of DEC‐205 expression in DC2.4 cells. (E) Flow cytometric analysis of DC2.4 cells treated with CFSE‐labelled mouse EVs and αDEC‐EVs (n = 4 biological repeats). (F) Flow cytometric analysis of DC2.4 cells treated with CFSE‐labelled human EVs and αDEC‐EVs (*n* = 3–4 biological repeats). (G) Western blot analysis and quantification of OVA protein loaded into OVA‐EVs and OVA‐αDEC‐EVs. (H) Representative flow cytometric plots and quantitative analysis of FITC‐OVA in DC2.4 cells treated with mouse EVs and αDEC‐EVs loaded with FITC‐OVA (*n* = 5–6 biological repeats). All graphs present mean ± SEM. *p < 0.05; ***p < 0.001, determined by one‐way ANOVA with Tukey's post‐hoc test.

DEC‐205 is a receptor expressed primarily in lymphoid DCs in T lymphocyte areas of lymphoid tissues and at lower levels on macrophages, monocytes, B cells and T cells.^24^ The important role of the DEC‐205 receptor in the cellular uptake of extracellular proteins and antigen processing is well‐documented. Targeted delivery of antigens via DEC‐205 receptor using antigen‐tagged antibodies has been shown to enhance the antigen presentation approximately 100‐fold more effectively than those delivered to DEC‐205^−^ DCs.^22^, ^25^ Here, to improve the antigen delivery and processing in DCs, we established a simple and efficient method for non‐covalent and orientation‐controlled conjugation of αDECab onto the RBCEV surface (Figure 2A). First, we used OaAEP1 ligase to create a covalent bond between the FcBP and surface proteins on the RBCEVs following our previous report.^15^ To facilitate the ligation, the Fc‐binding region (TCWVLEGLHWACD) was modified with the ligase binding site (NGL) and linker sequence (GGGGS) at the C terminus. The resulting FcBP‐conjugated EVs were then incubated with an αDECab to allow the attachment of the αDECab to the EVs via the Fc‐binding cyclic region of FcBP (DEC‐targeting EVs, αDEC‐EVs).^26^, ^27^ The amount of conjugated αDECab was quantified by Western blot and compared with a serial dilution of αDECab standard. The quantification showed that there was ~1.395 ng of αDECab per 1 μg of EVs, with each EV particle containing an estimated average of 40 anti‐DEC205 antibody molecules on average (Figure 2B). The size, zeta potential and polydispersity index (PDI) of αDEC‐EVs were not significantly different from non‐conjugated EVs (Figure 2C), suggesting a good compatibility of the established antibody ligation method.

Using flow cytometry analysis, we then evaluated the uptake of human and mouse RBCEVs by murine DC2.4 cells that highly express DEC‐205 (Figure 2D). We labelled EVs and αDEC‐EVs with CellTrace CFSE and treated DC2.4 cells for 2 h. As shown in Figure 2E, the percentage of CFSE‐positive DC2.4 cells treated with αDEC‐EVs was two‐fold higher than those treated with EVs (21.9% vs. 9.2%), indicating that the ligation with αDECab improved the uptake of EVs into DC2.4 cells via DEC‐205 receptor‐mediated endocytosis. We also employed our surface modification method for conjugating human EVs with αDECab and tested cellular uptake of human EVs by DC2.4 cells. Consistently, we observed a better internalisation of human αDEC‐EVs by DC2.4 cells, compared to control EVs (Figure 2F). In addition, we found that murine EVs were taken up more readily by the DC2.4 cells than human EVs, suggesting that the RBCEVs might be suitable carriers for administration into the cells from the same species.

We employed the same loading method as aforementioned to load OVA protein onto surface‐modified RBCEVs and quantified the amount of loaded OVA proteins. Western blot analysis showed that there was ~2.3 ng of OVA protein per 1 μg of RBCEVs (Figure 2G). We performed an additional purification step to completely remove unloaded OVA from EVs, using size‐exclusion chromatography column (SEC). Figure 2G indicates that the combination of centrifugation and SEC method could remove unattached OVA protein from EVs more efficiently. Similar amounts of OVA protein loaded into non‐modified and modified EVs confirmed that the surface modification of EVs did not interfere with the antigen‐loading process. It was noted that the antigen loading increased the size of αDEC‐EVs (from ~159.0 nm to ~192.3 nm as shown in Figure 2C). No EV aggregation was observed, and there was no significant change in the zeta potential of EVs during the OVA loading process.

We then used FITC‐OVA protein to investigate the protein delivery efficiency of OVA‐loaded surface‐functionalised RBCEVs in DC2.4 cells. The delivery efficiency was measured by quantifying the percentage of FITC‐positive cell population after 24 h of incubation with OVA‐loaded EVs. The delivery efficiency of OVA protein by αDEC‐EVs increased from 20.8% to 25.5% compared with non‐targeted EVs (Figure 2H), indicating that our targeting strategy enhanced both cellular uptake and protein transfection of RBCEVs in DCs. As we did not observe cell death during the experiment, OVA‐loaded EVs were safe for the cells in the in vitro setting. These data suggest that our loading method was suitable for loading OVA protein onto EVs while retaining the EV size, zeta potential and biocompatibility. Notably, treating DC2.4 cells with the combination of free OVA protein, RBCEVs, and free αDECab did not increase FITC signals in DC2.4 cells, indicating the crucial role of the EVs as a delivery platform of antigens. However, in our settings, the difference between targeted and non‐targeted EVs in protein delivery after 24 h was not statistically significant (*p* = 0.1195). As RBCEVs can be endocytosed by various pathways,^28^ we hypothesised that our RBCEVs could be targeted to DEC‐205 and were preferred to be uptaken via DEC‐205‐mediated endocytosis, at the expense of other pathways. An increase in DEC‐205‐mediated endocytosis of protein antigens could drive the antigen processing towards MHC‐I antigen presentation.

### 2.3 Targeted RBCEVs improved antigen presentation on MHC class I on DC2.4 cells

**FIGURE 3:**
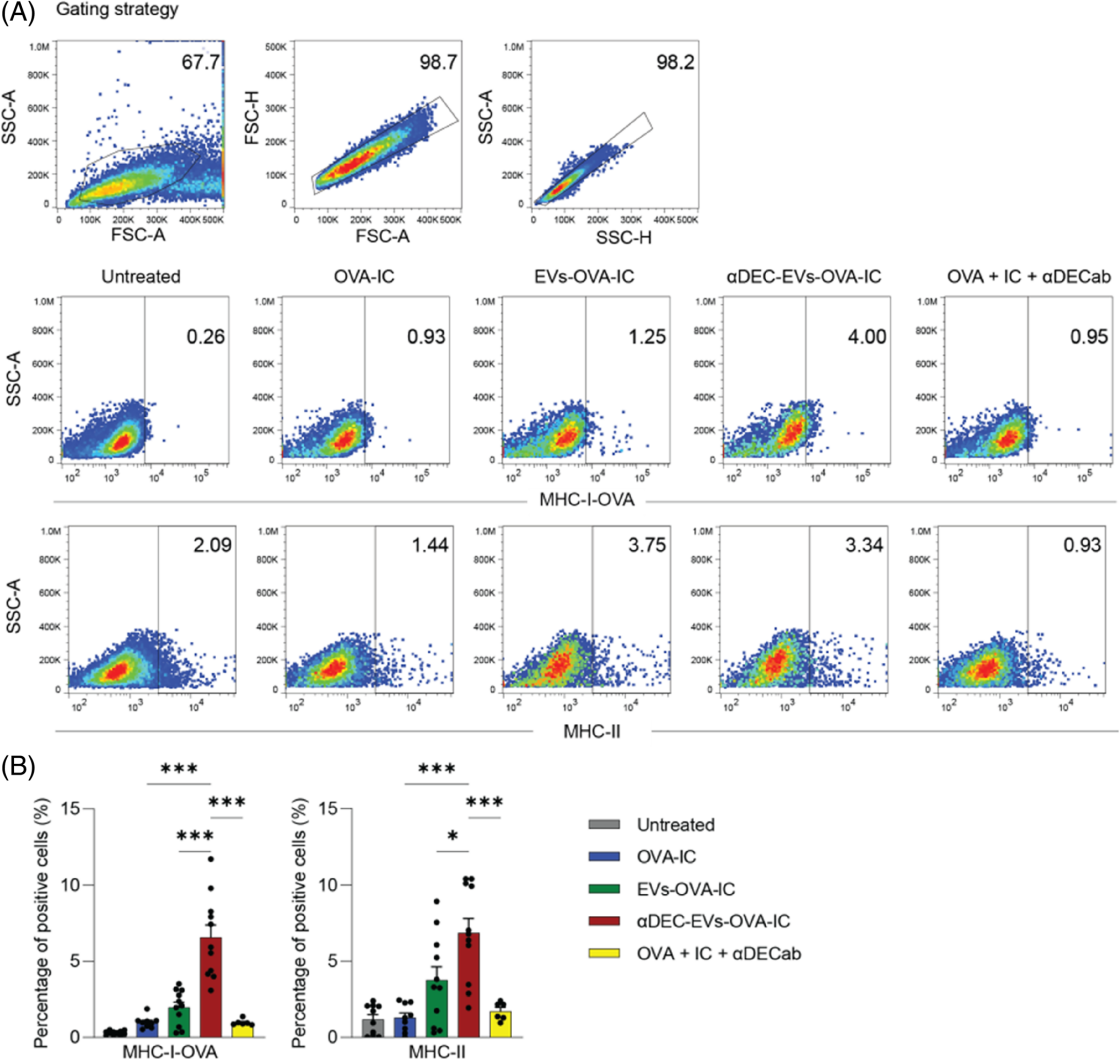
Targeted delivery of antigen by red blood cell‐derived extracellular vesicles (RBCEVs) enhanced antigen presentation by DC2.4 cells. (A) Representative flow cytometric analysis of MHC‐I‐OVA and MHC‐II levels in DC2.4 cells at 24 h after treatment with OVA‐loaded EVs or αDEC‐EVs. Samples with free OVA protein or with a combination of free OVA protein and free αDECab were included as negative controls. All samples contained poly(I:C) as a DC immunoadjuvant. (B) Percentage of DC2.4 cells positive for MHC‐I‐OVA and MHC‐II at 24 h after treatment with various conditions listed in (A). All graphs represent mean ± SEM, ns: not significant, *p < 0.05 and ***p < 0.001 determined by one‐way ANOVA with Tukey's post‐hoc test.

In this study, we co‐administered the antigen‐loaded EVs with poly(I:C), the most common immunoadjuvant for DC‐targeted vaccines via DEC‐205 receptor,^29^ to break the immune tolerance and augment a potent and long‐lasting anticancer immune response. We evaluated levels of OVA peptide (SIINFEKL) bound to MHC‐I of DC2.4 cells after treatment with EVs and poly(I:C) using flow cytometry. As shown in Figure 3A,B, the percentage of DC2.4 cells expressing MHC‐I‐OVA after αDEC‐EVs‐OVA treatment was 3‐fold higher than those that received EVs‐OVA treatment (6.7% vs. 2.0%). MHC‐II molecule was also elevated in DC2.4 cells treated with αDEC‐EVs compared with the non‐targeted EVs‐treated group (6.8% vs. 3.8%). Interestingly, free αDECab did not activate DCs, as indicated by similar expression levels of MHC‐II in free antibody‐treated DC2.4 cells compared to the untreated control. These data suggested that targeted antigen delivery via DEC‐205 receptor could induce a superior antigen presentation on MHC‐I and upregulation of MHC‐II on DCs in the presence of a DC‐activating adjuvant.

### 2.4 DC2.4 cells treated with antigen‐loaded RBCEVs enhanced T cell proliferation and T cell activation in a co‐culture system

**FIGURE 4:**
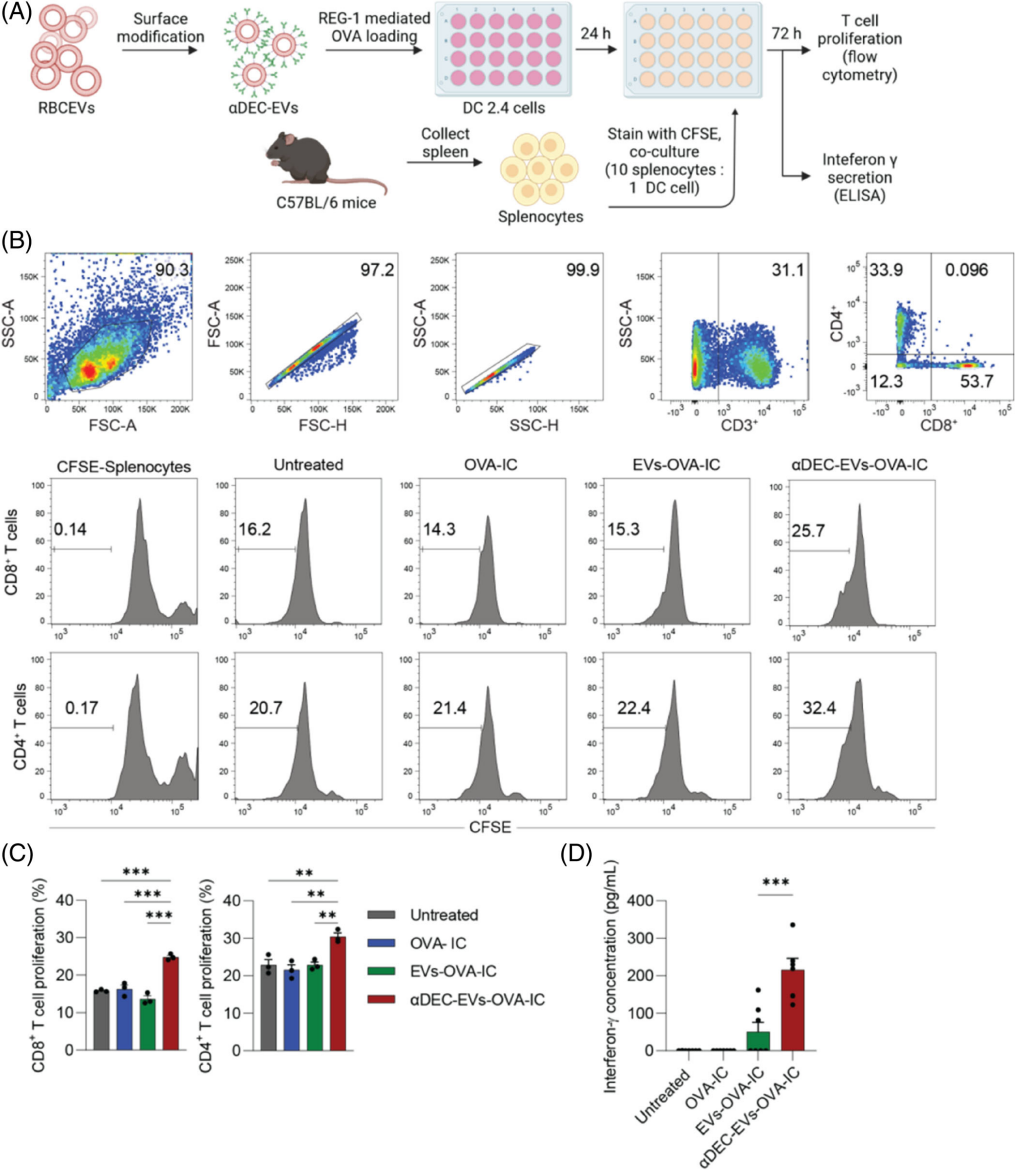
Targeted delivery of antigen by red blood cell‐derived extracellular vesicles (RBCEVs) improved T cell activation in DCs: splenocytes co‐culture system. (A) Schematic of experimental design of a DC: splenocyte co‐culture system. DC2.4 cells were pre‐treated with either OVA‐loaded EVs or αDEC‐EVs in the presence of poly(I:C) as DC immunoadjuvant for 24 h, then co‐cultured with CFSE‐labelled mouse splenocytes for 72 h before analysis. (B) Flow cytometric analysis of CFSE indicating T cell proliferation in the co‐culture system. Total T cells were gated from CFSE‐labelled splenocytes based on CD3^+^ markers, CD4^+^ and CD8^+^ T cells were gated based on their respective markers. Free OVA protein treatment was included as negative control for the experiment, and CFSE‐labelled splenocytes without co‐culture were included as a CFSE‐positive control. (C) Percentage of proliferating T cells quantified based on CFSE using flow cytometric analysis. (D) ELISA analysis of interferon‐γ level in the supernatant of the co‐culture system of each treatment in (C). All graphs represent mean ± SEM. ns: not significant, **p < 0.01; ***p < 0.001 determined by one‐way ANOVA with Tukey's post‐hoc test.

To investigate how EVs‐treated dendritic cells regulate the induction of T cell proliferation and activation, we co‐cultured EVs‐treated DC2.4 cells with CFSE‐labelled splenocytes isolated from naïve C57BL/6 mice. Total splenocytes from immunocompetent mice were used in our experiments to reflect the actual immunoactivity. A 1:10 ratio of DC: splenocyte was selected based on previous studies which demonstrate how DCs support T cell proliferation and activation.^30^ T cell proliferation was assessed by examining the appearance of cell sub‐populations bearing low CFSE signals as the T cells were activated and allowed to proliferate over time. T cell activation was determined by quantifying the secreted interferon gamma (IFN‐γ) concentration in the supernatant (Figure 4A). Flow cytometric analysis revealed that CD4^+^ and CD8^+^ T cells account for 33.9% and 53.7% of total CD3^+^ cells, respectively (Figure 4B). We observed the highest proliferation rate of both CD4^+^ and CD8^+^ T cells treated with αDEC‐EVs‐OVA in the presence of poly(I:C) when compared with control and non‐targeted EVs‐OVA (Figure 4B,C).

We further measured the concentration of IFN‐γ, a critical cytokine for innate and adaptive immunity,^31^ in the supernatant after co‐culturing splenocytes with treated DCs for 72 h. Figure 4D showed that the levels of IFN‐γ in samples treated with either EVs‐OVA‐IC or αDEC‐EVs‐OVA‐IC were quantifiable. In contrast, those of other samples treated with a combination of free poly(I:C)/OVA protein/αDECab or free OVA protein/poly(I:C) were not detectable. Notably, IFN‐γ levels from the αDEC‐EVs‐OVA‐IC‐treated group were much higher than those treated with EVs‐OVA‐IC (215.8 ± 75.61 pg/mL and 50.36 ± 67.08 pg/mL, respectively). These findings indicate that targeting DEC‐205^+^ DCs using αDECab‐modified RBCEVs leads to significantly enhanced T cell priming, which is crucial for effective cancer immunotherapy.

### 2.5 Conjugation with αDEC antibody increased the accumulation of RBCEVs in the spleen

**FIGURE 5:**
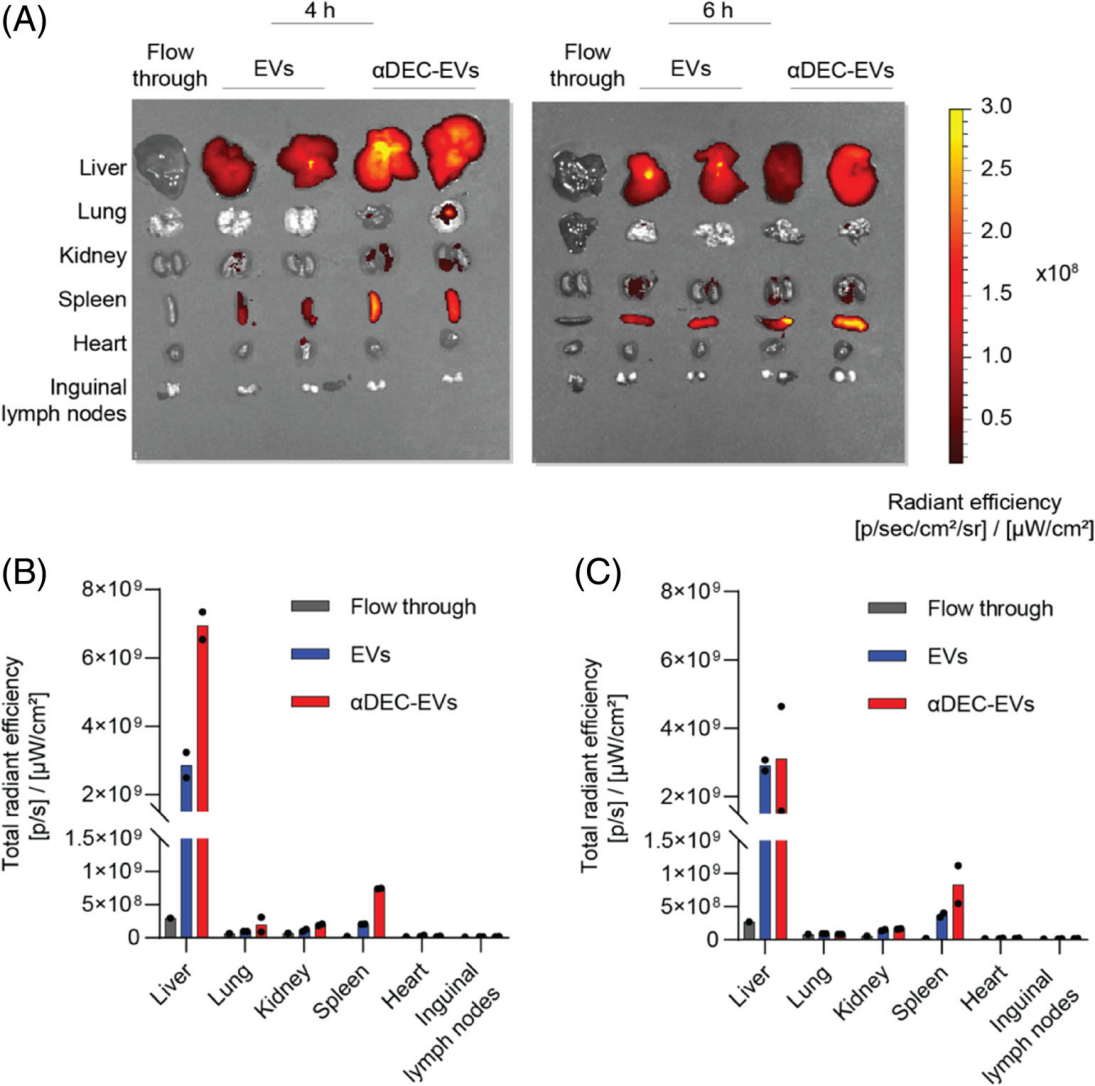
Conjugation with αDECab enhanced accumulation of red blood cell‐derived extracellular vesicles (RBCEVs) in the spleen. (A) Representative fluorescent images of organs from C57BL/6 mice administered with DiR‐labelled RBCEVs by i.p. injection. Images were captured 4–6 h after the injection using the IVIS. Colour scale represents the DiR signals. (B,C) Total radiant efficiency of DiR fluorescence in various organs of C57BL/6 mice at (B) 4 h and (C) 6 h post‐injection (*n* = 2 biological repeats).

To further support our findings with in vivo evidence, we intraperitoneally administered C57BL/6 mice with DiR‐labelled EVs and αDEC‐EVs. At different time points after the injection, we collected the major organs and analysed the DiR fluorescent signal using an in vivo imaging system (IVIS). Interestingly, αDEC‐EVs accumulated the most in the spleen of treated mice at both 4 h and 6 h post‐injection (Figure 5), suggesting their potential in specific delivery of antigens to the splenic DEC‐205^+^ DCs.

## 3 DISCUSSION

Cancer vaccines augment a specific antitumor immune response via DC activity, thus, enhancing specific antigen delivery and processing in DCs is crucial for effective cancer vaccination. The DEC‐205 receptor is highly expressed by cortical thymic epithelial cells and some DC subsets, such as the splenic CD8+ DCs that cross‐present antigens from apoptotic cells. Antigens that are endocytosed through DEC‐205 access both MHC‐I and MHC‐II antigen presentation pathways and targeting antigens to DEC‐205 with antibodies enhances T cell immunity for vaccination.^22^

Several EV types had been utilised to stimulate DCs towards antitumor activity, including but not limited to DC‐derived EVs, engineered HEK‐293 cell‐derived EVs and MSC‐derived EVs.^32^, ^33^, ^34^ To translate these findings into relevant industrial applications, certain challenges must be addressed. Primary cell line‐derived EVs such as DC‐EVs or MSC‐EVs suffer from the limitation of small‐scale production and tedious processing steps,^35^ while immortalised cell line‐derived EVs like HEK‐293 cell‐EVs or tumour‐derived EVs may be at risk of transferring oncogenic DNA and protein contamination.^36^, ^37^ RBCEVs, on the other hand, can be produced at a large scale in an industrial setting and have a good safety profile suitable for clinical applications.^14^

Here, we aim to develop a targeting strategy to increase antigen delivery to DEC‐205^+^ DCs using the RBCEV platform, thereby enhancing the potency of both cellular and humoral immune responses against cancer. In our project, we modified the RBCEV surface with Fc binding peptide and αDECab to enhance EVs' binding onto DCs and directed EVs to DEC‐205‐mediated endocytosis. FcBP is a small cyclic amino acid sequence specifically targeting the Fc region of IgG antibody molecules.^26^ To facilitate enzymatic ligation, we designed an FcBP fused with a linker peptide and NGL sequence at the C‐terminal. The amino acid sequences of the linker peptides determine the flexibility, conformation, hydrophilicity and spatial distance.^38^ Therefore, we chose the most common flexible sequence GGGGS as our linker peptide. The ligation of FcBP onto RBCEVs provided a robustness to our platform, as it could be easily altered to target different surface receptors by changing the antibody during the conjugation step. Combining the FcBP ligation with the antibody conjugation process demonstrated a gentle surface modification method suitable for RBCEV functionalisation for targeted therapy. Following surface modification, the cellular uptake of RBCEVs by DC2.4 cells was investigated. Cellular uptake of targeted EVs increased twice compared to the uptake of non‐targeted EVs regardless of species of EV origins which implied that surface modification of RBCEVs could improve cellular uptake. However, other factors could influence this process, such as the steric hindrance that reduces access to DEC‐205 antibodies or the number of DEC‐205 antibodies per EV.

As we focus on the antitumor effect of EV‐treated DCs, antigen presentation on MHC‐I and activation of cytotoxic T cells become important indications of a robust immune response against neoantigens. Our data show that the delivery efficiency of protein antigens using RBCEVs was the highest among all tested antigens; hence we proceeded with OVA protein as an antigen candidate. We proved that RBCEVs could be used as an effective carrier for protein delivery to recipient cells. The presence of RBCEVs in the transfection mix minimised cellular toxicity of loading agents, improved MHC‐II presentation on DCs, and increased T cell activation. Even though the antigen presentation on MHC‐I and MHC‐II on DC2.4 was only 5%, RBCEVs‐treated DC2.4 cells could trigger a robust T cell activation and proliferation. Our method for surface modification, targeting strategy, and OVA protein loading onto EVs could generate functional RBCEVs to stimulate DC maturation, antigen presentation onto MHC‐I, and subsequently trigger CD8^+^ T cell activation. The platform is robust for use in other applications, as we can easily change the targeting antibody to direct drug‐carrying EV towards different cell types.

## 4 MATERIALS AND METHODS

### 4.1 Cell culture

Mouse DC2.4 dendritic cell line was a kind gift from Prof. Lim Hsu Kim's lab (National University of Singapore). Blood samples from healthy donors were collected with their informed consent under the Singapore Health Science Authority guidelines. Human peripheral blood mononuclear cells (PBMCs) were isolated from these samples using the Ficoll‐Paque gradient method described in our previous report.^17^ PMBCs and DC2.4 cells were maintained in RPMI (Thermo Fisher Scientific, USA) supplemented with 10% foetal bovine serum (FBS) (Biosera, France), 1% penicillin–streptomycin (pen‐strep) (Thermo Fisher Scientific, USA), and 5 μg/mL Plasmocin prophylactic (InvivoGen, USA) in an incubator at 37°C with 5% CO_2_.

To generate immature human cDCs, freshly isolated monocytes were plated on 24‐well polystyrene plates at the concentration of 100,000 cells/well and maintained in RPMI supplemented with 10% FBS, 1% pen‐strep, 5 μg/mL plasmocin prophylactic, human GM‐CSF (80 ng/mL) and human IL‐4 (100 ng/mL) (Peprotech, USA) for 6 days. For mature DCs, after differentiation, the cells were matured with human TNFα (10 ng/mL), IL‐1β (10 ng/mL), IL‐6 (10 ng/mL) (Miltenyi Biotec, USA) and PGE2 (1 μg/mL) (Sigma‐Aldrich, USA).

### 4.2 Purification and characterisation of RBCEVs

Mouse blood was collected from C57BL/6 mice by either submandibular blood collection or cardiac puncture and mixed with anticoagulant Citrate Phosphate Double Dextrose (CP2D) solution containing 51.1 g/L glucose, 2.22 g/L mono sodium phosphate, 26.3 g/L sodium citrate and 3.27 g/L citric acid, then strained through a 40 μm cell strainer to remove blood clots. Mouse whole blood was centrifuged at 1200 ×*g* for 8 min at 4°C to remove non‐RBC components such as plasma, white blood cells, and platelets. Obtained RBCs were washed twice with sterile PBS, then resuspended in Additive Solution‐7 (AS‐7) buffer containing 3 μM calcium ionophore, 800 μM calcium chloride at the concentration of 4 × 10^9^ RBCs/mL and incubated at 37°C for RBCEV induction. After 4 h, the EV induction solution was collected and subjected through sequential centrifugation at 1600 ×*g* and 4000 ×*g* for 10 min to remove cell debris. The supernatant was then pooled and filtered through 0.45 μm filter and centrifuged at 21000 ×*g* for 1 h. RBCEV pellets were finally washed with PBS twice at 21000 ×*g*, 30 min, and resuspended in PBS for downstream application. For long‐term storage, RBCEVs were resuspended in PBS with 4% (w/v) trehalose and stored at −80°C.

Human RBCEVs were purified from group O human RBCs. Blood samples from healthy donors with informed consent were kindly provided by Singapore Health Sciences Authority and Hong Kong Red Cross. Blood cells were separated from plasma by centrifuging at 1000 ×*g* for 8 min at 4°C and washed thrice with PBS. White blood cells were removed from total blood cells by leukoreduction filter (Nigale, China). The harvested RBCs was resuspended in Nigale buffer and diluted with 0.1 mg/mL calcium chloride and 10 μM of calcium ionophore (Sigma‐Aldrich, USA) in PBS buffer and incubated overnight at 37°C, 5% CO_2_. RBCEVs were then purified using our established protocol as described previously.^18^ For long‐term storage, RBCEVs were resuspended in PBS containing 4% (w/v) trehalose and stored at −80°C. To quantify RBCEVs for downstream application, haemoglobin content was used as an indicator and measured by Nanodrop 2000 instrument (Thermo Fisher Scientific, USA).

### 4.3 Characterisation of RBCEVs

Particle size, concentration and zeta potential of RBCEVs were measured by a particle tracking analysis system (ZetaView®, Particle Metrix, Germany). For size and particle concentration measurement, RBCEVs were diluted 10,000 times in filtered PBS. For zeta potential measurement, RBCEVs were diluted in filtered 10 mM HEPES buffer prior to analysis. For general quantification of RBCEVs throughout the project, their total haemoglobin content was used as an indicator. Haemoglobin content was quantified by measuring absorbance of RBCEV solution at isosbestic points (420 nm and 586 nm) for Oxy‐Hb/Hb, using Nanodrop 2000 instrument (Thermo Fisher Scientific, USA).

### 4.4 Surface modification and antibody conjugation of RBCEVs

Fc‐binding peptide TCWVLEGLHWACDGGGGSNGL was designed to contain a functional domain NGL at the C‐terminal to facilitate enzymatic ligation to RBCEV surface proteins.^38^ The sequence of Fc‐binding region was obtained from the work of Shim et al.^26^ Peptides were synthesised and provided by GL Biochem Ltd., Shanghai, China and resuspended in PBS before use.

For enzymatic ligation, RBCEVs were incubated for 1 h at 25°C in a solution containing 500 μM FcBP and 2 μM OaAEP1 ligase in PBS at pH 7. Following ligation, RBCEVs were washed three times with PBS at 21,000 ×*g* for 20 min at 4°C to remove free peptides. FcBP‐ligated RBCEVs (Fc‐EVs) were incubated with 2 μg/μL anti‐mouse DEC‐205 antibody for 2 h on a gentle shaker, followed by three washes with PBS at 21,000 ×*g* for 20 min at 4°C to remove free antibodies. αDECab‐conjugated RBCEVs were resuspended in PBS for further analysis.

### 4.5 Protein loading onto RBCEVs

Ovalbumin (OVA) protein (Sigma‐Aldrich, USA) was incubated with REG‐1 loading agent for 15 min at room temperature (RT) to form transfection complex. The complex was mixed with RBCEVs at a ratio of 8 μg of OVA protein: 50 μg of RBCEV in PBS for 30 min at 25°C on a gentle mixer. OVA‐loaded RBCEVs were pelleted by centrifugation at 21,000 ×*g*, 20 min, 4°C, then resuspended in PBS and subjected to size exclusion chromatography (SEC) to remove free OVA protein and free OVA‐REG‐1 complex. Loaded RBCEVs were pelleted at 21,000 ×*g*, 20 min, 4°C, and resuspended in PBS.

### 4.6 Western blot analysis

Characterisation of RBCEVs, antibody conjugation, and OVA protein loading efficiency was determined by Western blot analysis. The concentration of loaded proteins was determined by haemoglobin content as described above. Mouse RBCs or RBCEVs were lysed in RIPA buffer (Thermo Fisher Scientific, USA) containing protease inhibitors (Biotool, USA) for 5 min on ice. 20 μg EV lysates were diluted in Laemmli buffer (Biorad, USA) supplemented with 10% β‐mercaptoethanol and heated at 95°C for 5 min. Electrophoresis was conducted at 120 V for 2 h until the sample dye reached the gel bottom. Proteins were transferred to a polyvinylidene difluoride (PVDF) membrane (Immobilon‐P, Millipore) using the wet transfer method. Thereafter, the membrane was blocked with 5% skimmed milk (BD Biosciences, USA) in Tris‐buffered saline supplemented with 0.1% Tween‐20 (TBST) for 1 h at RT. To detect GAPDH, CD9, Alix and TSG101, the membrane was incubated with anti‐CD9 (Abcam, USA), anti‐GAPDH, anti‐Alix and anti‐TSG101 (Santa Cruz, USA) at 1:1000 dilution overnight, washed thrice with TBST then incubated with anti‐mouse antibody (Vector Laboratories, UK) at dilution of 1:10.000 for 1 h at RT. To detect and quantify OVA protein, the membrane was incubated with anti‐OVA antibody (Abcam, USA) at 1:3000 dilution for 2 h at RT, washed thrice with TBST then incubated with anti‐rabbit antibody (Vector Laboratories, UK) at dilution of 1:10.000 for 1 h at RT. To detect and quantify αDECab, the membrane was incubated with anti‐rat antibody (Vector Laboratories, UK) at a dilution of 1:10.000 for 1 h at RT. The membrane was washed five times with TBST to remove all traces of secondary antibody. The signals were developed with the WesternBright ECL HRP substrate kit (Advansta, USA) and detected by ChemiDoc XLS+ Imaging System instrument (BioRad, USA). Full images of the blots are provided in Figure S1. The intensity of the bands were quantified using ImageJ.

### 4.7 Cellular uptake of RBCEVs

DC2.4 mouse dendritic cells were seeded into 48‐well plate at a density of 25,000 cells/well overnight. On the next day, 25 μg of EVs or αDEC‐EVs were labelled with Cell Trace® CFSE for 2 h at 37°C on a gentle shaker in the dark, wash twice with PBS, then added into DC2.4 cells and incubated at 37°C, 5% CO_2_. For flowthrough control, CFSE‐labelled EVs were incubated in PBS at 37°C for an additional 2 h, then centrifuged at 20,000 ×*g* for 20 min and supernatant were added into DC2.4 cells. After 2 h, cells were trypsinised, washed with PBS two times, and subjected to flow cytometric analysis using BD LSR Fortessa™ Cell Analyzer instrument (BD Biosciences, USA).

### 4.8 Transfection efficiency of OVA protein into dendritic cells

DC2.4 cells were seeded into 48‐well plate at a density of 25,000 cells/well overnight. On the next day, 25 μg of EVs or αDEC‐EVs were loaded with FITC‐OVA protein, added into DC2.4 cells, and incubated for 24 h at 37°C, 5% CO_2_. Cells were trypsinised, washed twice with PBS and analysed by flow cytometry using BD LSR Fortessa™ Cell Analyzer instrument (BD Biosciences, USA).

### 4.9 Antigen presentation of DC2.4 cells

DC2.4 cells were seeded into 48‐well plate at a concentration of 25,000 cells/well overnight. On the next day, 25 μg of EVs or αDEC‐EVs loaded with OVA protein, 250 ng/mL of OVA protein, and/or 500 ng/mL of Polyinosinic‐polycytidylic acid (Poly(I:C)) (Invivogen, USA) were added into DC2.4 cells and incubated at 37°C, 5% CO_2_. After 24 h, cells were trypsinised, washed with PBS, stained with APC anti‐mouse H‐2K^b^ bound to SIINFEKL, PE anti‐mouse I‐A/I‐E, APC/Cyanine7 anti‐mouse CD86 antibodies (Biolegend, USA) and subjected to a flow cytometer (BD LSR Fortessa™ Cell Analyzer, BD Biosciences, USA).

### 4.10 Isolation of mouse splenocytes

Spleen was harvested from naïve 6–8 weeks old C57BL/6 mice and macerated through a sterile 70 μM cell strainer (BD Biosciences, USA) to release splenocytes. The cells were washed with ice‐cold RPMI supplemented with 2% FBS by centrifugation at 500 ×*g* for 5 min at 4°C. RBCs were lysed by adding 1 mL of RBC lysis buffer (Gibco, USA) to the cell supernatant and incubated at room temperature for 3 min. After adding 4 mL of ice‐cold RPMI supplemented with 10% FBS to stop the reaction, the cells were washed with PBS and counted using Countess II FL Automated Cell Counter instrument (Thermo Fisher Scientific, USA). Splenocytes were stained with CFSE using CellTrace™ CFSE Cell Proliferation Kit (Thermo Fisher Scientific, USA) following the manufacturer's guideline.

### 4.11 Dendritic cell—Splenocyte co‐culture

DC2.4 cells were seeded into 48‐well plates at a density of 25,000 cells/well overnight. On the next day, 25 μg of EVs or αDEC‐EVs loaded with OVA protein, 250 ng/mL of OVA protein, and/or 500 ng/mL of Polyinosinic‐polycytidylic acid (Poly(I:C)) (Invivogen, USA) were added into DC2.4 cells and incubated at 37°C, 5% CO_2_. After 24 h, the supernatant was removed, then CFSE‐labelled splenocytes were added into the wells at the DC:splenocyte ratio of 1:10 and incubated at 37°C, 5% CO_2_. After 72 h, cells in suspension were collected from the plate, washed twice with PBS at 300 ×*g*, 10 min, and stained with APC anti‐mouse CD8a, PE anti‐mouse CD3e, BV421 anti‐mouse CD4 antibodies (Biolegend, USA). CD3^+^CD4^+^ and CD3^+^CD8^+^ cell proliferation was determined by flow cytometry using BD LSR Fortessa™ Cell Analyzer instrument (BD Biosciences, USA). T cell activation was determined by measuring the concentration of secreted IFN‐γ in the cell supernatant using ELISA MAX™ Deluxe Set Mouse IFN‐γ kit (Biolegend, USA).

### 4.12 Biodistribution of targeted‐RBCEVs in immunocompetent mice

Animal studies were conducted following the protocols approved by the Institutional Animal Care and Use Committee at National University of Singapore (NUS). C57BL/6 mice were provided by the Jackson Laboratory (USA). To determine the biodistribution of RBCEVs, EVs or αDEC‐EVs labelled with DiR (1,10‐Dioctadecyl‐3,3,30,30‐Tetramethylindotricarbocyanine Iodide) dye and flow‐through control were injected into the mice through i.p. injection. After 4 h or 6 h, mice were euthanised and main organs were collected to analyse DiR signals using the IVIS Lumina III system (PerkinElmer, USA).

### 4.13 Statistical analysis

Student's one‐tailed *t*‐tests were performed using GraphPad Prism (Graphpad Software, USA) to analyse significant differences between treated and control groups. One‐way ANOVA, computed using GraphPad Prism 9, were used to analyse the differences between treated groups with Tukey's post‐hoc test. P‐value of less than 0.05 was considered statistically significant, based on at least three independent replicates. Data are presented as the mean ± standard error of the mean.

## AUTHOR CONTRIBUTIONS

X.T.T.D., C.D.P. and M.T.N.L. conceived of the presented idea. X.T.T.D. and C.D.P. designed the experiments, analysed the data and designed figures. X.T.T.D., C.D.P., C.M.H.L. and J.A. performed the experiments and collected the data. M.K.J. provides resources and technical support. X.T.T.D. and C.D.P. wrote the manuscript in consultation with T.T., H.S. and M.T.N.L. C.M.H.L., T.T., H.S. and M.T.N.L. contributed to text and the content of the manuscript, including revision and edits. M.T.N.L. supervised the project. All authors read and approved the final manuscript.

## FUNDING INFORMATION

This project is supported by the Ministry of Health (NMRC/OFIRG/MOH‐000643), the National University of Singapore (grant number NUHSRO/2019/076/STARTUP/02) and Carmine Therapeutics collaboration grant.

## CONFLICT OF INTEREST STATEMENT

Minh TN Le is a scientific co‐founder, advisor and shareholder of Carmine Therapeutics, a company that develops extracellular vesicle‐based therapy. Other authors declare no conflict of interest.

## Supporting information


**FIGURE S1.** Full images of the Western blots. (A) Western blot analysis of OVA protein standard amounts (lane 1 to 5, corresponding to 250, 125, 62.5 and 31.25, and 15.625 ng of OVA, respectively), and OVA protein loaded into EVs (lane7), αDEC‐EVs (lane 8) and EVs before SEC (lane 9). (B) Western blot analysis of αDECab standard amounts (lane 1 to 5, corresponding to 250, 125, 62.5, 31.25 and 15.625 ng of αDECab, respectively) and αDECab conjugation onto RBCEVs (lane 6), showing bands corresponding to both heavy chain (50 kDa) and light chain (25 kDa). (C) Western blot analysis of Alix, TSG101, GAPDH, CD9, β‐tubulin and Ter119 in RBC lysates (lane 1) and RBCEVs (lane 2).

## Data Availability

The data supporting the findings of this study are available as part of the article and its supplementary material and no additional source data are required.
