# Supplementary material for: Dendritic cell‐targeted delivery of antigens using extracellular vesicles for anti‐cancer immunotherapy
Source: Cell Prolif. 2024 Mar 20;57(7):e13622. doi: 10.1111/cpr.13622 (PMC11216926; doi:10.1111/cpr.13622)
Supplement: Supplementary file 1 — FIGURE S1. Full images of the Western blots. (A) Western blot analysis of OVA protein standard amounts (lane 1 to 5, corresponding to 250, 125, 62.5 and 31.25, and 15.625 ng of OVA, respectively), and OVA protein loaded into EVs (lane7), αDEC‐EVs (lane 8) and EVs before SEC (lane 9). (B) Western blot analysis of αDECab standard amounts (lane 1 to 5, corresponding to 250, 125, 62.5, 31.25 and 15.625 ng of αDECab, respectively) and αDECab conjugation onto RBCEVs (lane 6), showing bands corresponding to both heavy chain (50 kDa) and light chain (25 kDa). (C) Western blot analysis of Alix, TSG101, GAPDH, CD9, β‐tubulin and Ter119 in RBC lysates (lane 1) and RBCEVs (lane 2). [file CPR-57-e13622-s001.docx]

**Supporting Information**

**Dendritic cell-targeted delivery of antigens using extracellular vesicles for anti-cancer immunotherapy**

Xuan TT Dang^1,2^, Cao Dai Phung^1,2^, Claudine Ming Hui Lim^1,2^, Migara Kavishka Jayasinghe^1,2^, Jorgen Ang^3^, Thai Tran^4,5,6^, Herbert Schwarz^4,6^, Minh TN Le ^1,2,6,7,8*^

^1^Department of Pharmacology, Yong Loo Lin School of Medicine, National University of Singapore, Singapore

^2^Institute for Digital Medicine, Yong Loo Lin School of Medicine, National University of Singapore, Singapore

^3^School of Applied Science, Republic Polytechnic, Woodlands, Singapore

^4^Department of Physiology, Yong Loo Lin School of Medicine, National University of Singapore, Singapore

^5^Infectious Disease Translational Research Program, National University of Singapore, Singapore

^6^Immunology Programme, National University of Singapore, Singapore, Singapore

^7^Department of Surgery, Yong Loo Lin School of Medicine, National University of Singapore, Singapore

^8^Institute of Molecular and Cell Biology, Agency for Science, Technology, Technology and Research, Singapore

Xuan TT Dang and Cao Dai Phung contributed equally to this work.

*Corresponding author.

*E-mail address:* phcltnm@nus.edu.sg (Minh TN Le).

**
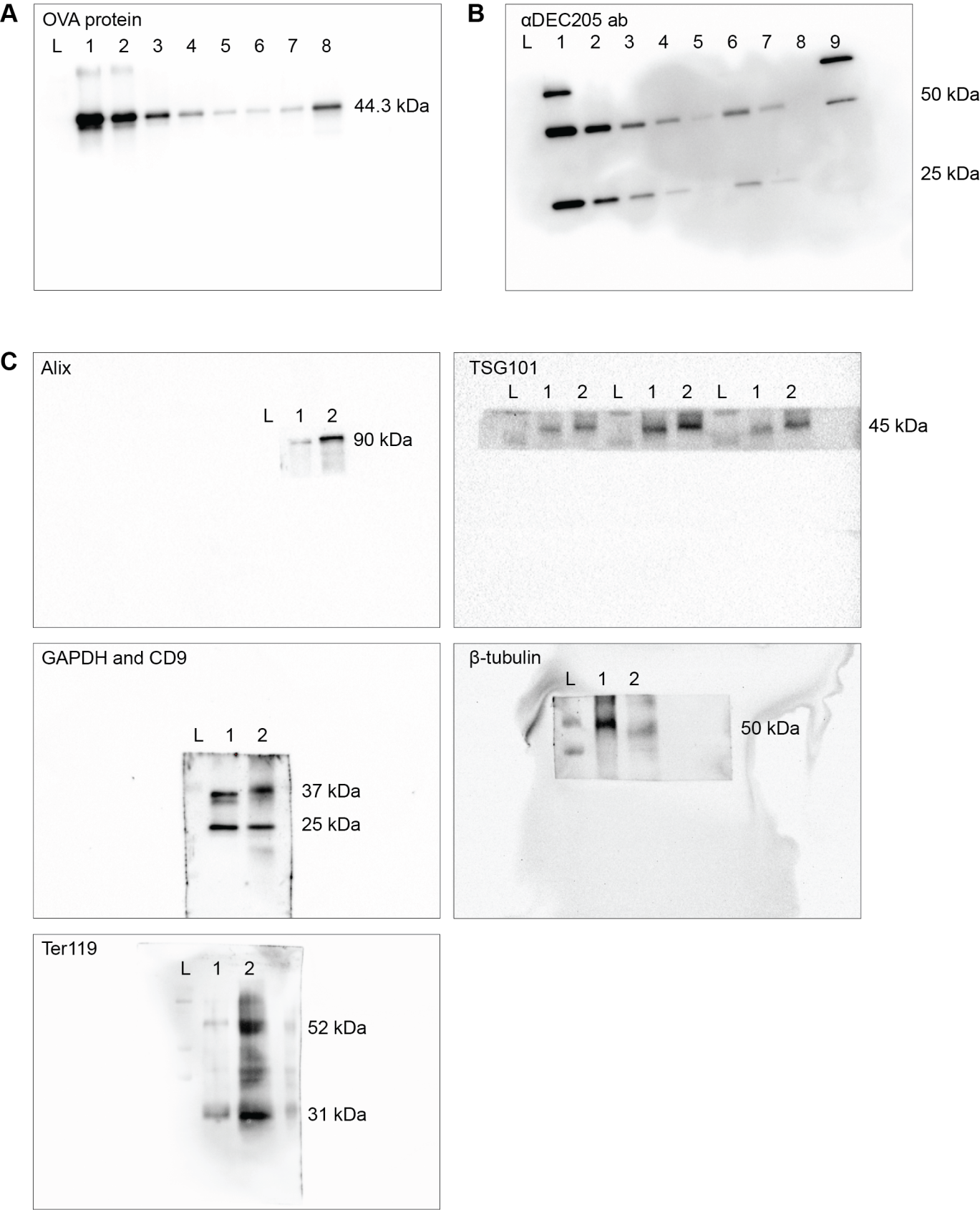
**

**Figure S1. Full images of the Western blots.** (**A**) Western blot analysis of OVA protein standard amounts (lane 1 to 5, corresponding to 250, 125, 62.5, and 31.25, and 15.625 ng of OVA, respectively), and OVA protein loaded into EVs (lane7), αDEC-EVs (lane 8) and EVs before SEC (lane 9). (**B**) Western blot analysis of αDECab standard amounts (lane 1 to 5, corresponding to 250, 125, 62.5, 31.25, and 15.625 ng of αDECab, respectively) and αDECab conjugation onto RBCEVs (lane 6), showing bands corresponding to both heavy chain (50 kDa) and light chain (25 kDa). (**C**) Western blot analysis of Alix, TSG101, GAPDH, CD9, β-tubulin and Ter119 in RBC lysates (lane 1) and RBCEVs (lane 2).
